# The short-term impact of terrorism on public mental health: an emergency primary care approach

**DOI:** 10.1186/s12889-023-17240-z

**Published:** 2023-11-24

**Authors:** Lisa Govasli Nilsen, Tore Wentzel-Larsen, Lise Eilin Stene

**Affiliations:** 1 Norwegian Centre for Violence and Traumatic Stress Studies, P.B. 181 Nydalen, 0409 Oslo, Norway; 2 Department of Sociology and Political Science, Norwegian University of Science and Technology, NTNU, Trondheim, Norway; 3 Centre for Child and Adolescent Mental Health, Eastern and Southern Norway, Oslo, Norway

**Keywords:** Terrorism, Disasters, Primary care, Mental health, Time series data, Healthcare utilisation

## Abstract

**Background:**

Terrorist attacks commonly have mental health consequences for those directly affected. Existing research is, however, divided when it comes to how and whether terrorist attacks affect the general population’s mental health. There is a need for studies investigating a broader range of mental health reactions to understand more about how different groups of the population are affected by terrorist attacks, while also illuminating important systemic factors.

**Methods:**

In this study we investigated whether there was any change in the number of consultations with out-of-hours emergency primary care for psychological reactions in association with the 2011 terrorist attacks in Norway. Data covering the entire Norwegian population’s primary care contacts in 2008–2013, where the reason for encounter was coded as psychological concerns or psychiatric disorders, were studied. A time series intervention analysis, using ARIMA modelling, was used to estimate whether there was indeed a change in healthcare utilisation associated with the terrorist attacks.

**Results:**

The analysis uncovered an increase in contacts with emergency primary care by the overall population for mental health concerns associated with the terrorist attacks. When divided into groups according to geographical proximity to attacks, no significant change was found in the area closest to the attack in Oslo, whereas an increase was found for the rest of the country. There was also heterogeneity across different age groups. An increase was found among youths, young adults, and middle-aged people, but not the other age groups, and an increase was found for both men and women.

**Conclusions:**

These findings highlight the need for primary care services to be prepared to meet mental health reactions in the general population when planning for healthcare provision in the aftermath of terrorism. Simultaneously, it should be noted that needs may vary across different groups of the population.

**Supplementary Information:**

The online version contains supplementary material available at 10.1186/s12889-023-17240-z.

## Highlights

• Terrorism increases general populations’ help-seeking for mental health concerns.

• Groups of the population are more affected than others.

**Supplementary Information:**

The online version contains supplementary material available at 10.1186/s12889-023-17240-z.

## Background

Terrorist attacks may induce fear, stress and a sense of insecurity in the general population [1–4]. An important question for research has been whether this fear is linked to adverse outcomes for health and wellbeing in the general public, in addition to what is observed in groups directly exposed to attacks. Additionally, it is important to scrutinize, whether such events imply more healthcare seeking for the population at large due to psychological disorders or concerns. This study investigates whether there was any change in contacts made with emergency primary care for psychological disorders or concerns in the immediate aftermath of the 22 July 2011 terrorist attacks in Norway.

Several studies have investigated health outcomes in the general population after terrorist attacks, such as following the Oklahoma bombings in 1995 [5], the 9/11 attacks in the United States in 2001 [6–8], the 2005 London attacks [4], and the 2004 Madrid attacks [6]. So far, this research portrays a somewhat mixed picture of whether and how terrorism affects populations beyond the ones directly exposed. Whereas some studies observe psychological or somatic reactions in the general population in the aftermath of terrorism [9–12], others do not [1, 13, 14], or they find diverging results across different attacks [6]. It remains unclear whether this inconclusiveness in the literature can be ascribed to characteristics of diverse populations and study contexts, or different methodologies across studies. More research is needed on whether reactions occur in diverse study contexts and populations. Furthermore, a stronger focus on the methodological approaches is needed to clarify if divergent findings could be due to differences in terms of how and when health outcomes are measured.

When health consequences in the general population are studied after terrorist attacks, the focus is typically on potentially stress-induced health concerns. This includes cardiovascular diseases (e.g., [9, 13, 15]), maternal-child health outcomes [9, 6], psychiatric diagnoses and suicides [9–11, 16] and psychological distress more broadly defined [1, 2, 4, 8, 17]. In general, studies either rely on self-reports of psychological reactions (e.g., [1, 2, 4, 8]), or registry data that include information on for instance diagnoses or deaths that could be stress-induced (e.g., [6, 9–13]). Much of existing research has focused on health consequences and diagnoses that typically will be detected and need treatment in specialized healthcare (e.g., [9–11, 13]). Hence, records from specialized healthcare providers are utilized to study the effects of terrorism. In countries with gatekeeping systems, access to mental health services (MHS) generally occurs through referral from primary care [18]. Many patients seeking care for psychological concerns will be treated in primary care without further referral, and those who are referred to specialized MHS will often have to wait before they access consultations within MHS. Therefore, it is important to study consultations with primary care services for psychological concerns, in order to capture potential acute changes in help seeking. The out-of-hours emergency primary care service (Legevakt) is an important service to scrutinize in this regard because it generally receives acute inquires. Furthermore, registry data from the healthcare system give information about different forms of healthcare utilization. This system aspect is also important to explore further, as healthcare systems vary across countries. Knowledge about if and where post-terror health reactions can be identified in the healthcare system is essential for a better understanding of systemic responses and future planning.

To our knowledge, there exist no studies that utilise pre- and post-terror registry data to study psychological disorders and reactions that are typically detected in primary care. The current study therefore applies pre- and post-attack registry data on the entire population to study utilisation of emergency primary care due to psychological concerns after a terrorist attack.

Studying more commonly occurring conditions enables us to investigate how groups of the population might be affected differently without breaching anonymity. Previous studies indicate that certain groups of the population may be more vulnerable to develop reactions in the aftermath of terror than others. Living in geographical proximity to terrorist attacks has been found to be associated with higher notions of distress [2, 11]. Following the 9/11 attacks for example, higher levels of post-traumatic stress reactions were found in New York City, compared to the rest of the country, particularly among residents living near the attack sites [19]. After the 22 July attacks in Norway, Thoresen et al. [2] found that fear responses and jumpiness were particularly high among the residents of Oslo, and that geographical closeness was associated with early emotional reactions.

At the same time, the mental health consequences of terrorist attacks have also been observed far away geographically, even in countries that are not directly affected by the attacks [8, 10, 11]. In two studies from Denmark, an increase in stress-related diagnoses was found in the general population, after both the 9/11 attacks in the United States in 2001 [11] and the terrorist attacks in Norway in 2011 [10], with a significantly larger increase following the Norway attacks. Experiencing psychological or social proximity to the attacks is another possible explanation, either through knowing someone directly affected [2], or through identifying with those directly affected in other ways [20]. Linked to this is the question of whether demographic or individual characteristics can lead groups of the population to identify more strongly with those directly affected, and be more prone to develop health reactions. While female sex is often found to be a predictor for mental health outcomes [19, 21], less is known about the sex distribution of mental healthcare utilization in the general population after terrorism. Age can also be another relevant demographic factor to account for, both because young age has been found to be a risk factor for certain mental health outcomes [19], but also because generational belonging can be an important factor for perceived closeness to directly affected groups.

Addressing the gaps in the literature outlined above, the main aim of this paper is to investigate the effect of a terrorist attack on the general population’s mental health. More specifically, the study has two objectives: First, to investigate whether there was any change in the number of contacts with out-of-hours emergency primary care (emergency primary care, from here on) for psychological reactions directly after the 2011 terrorist attacks in Norway. Second, to determine whether different groups of the population in terms of age, sex and geographical closeness to the attacks were affected differently.

### Hypotheses

Based on the existing literature outlined above, the following hypotheses were formulated:

- H1: The terrorist attacks were associated with an immediate, short-term increase in consultations with emergency primary care, regarding psychological concerns and disorders.
- H2: The increase in healthcare contacts was more elevated in the capital Oslo, as compared to the rest of the country.

Given that existing research, as outlined above, is limited when it comes to how sub-groups of the population are affected heterogeneously by terrorist attacks, there was no predefined expectation concerning the role of the patient’s individual characteristics (age and sex).

## Methods

### Study context

The terrorist attacks under scrutiny are the 22 July 2011 terrorist attacks in Oslo and on Utøya Island in Norway. In these attacks, one perpetrator, with sympathies to the extreme right, first launched a bomb at the governmental buildings in Oslo, killing eight people and injuring several others. He then travelled to the island of Utøya, about 40 km outside of Oslo, where the Norwegian Labor Youth were holding their annual summer camp. Dressed as a police officer, the perpetrator committed a shooting spree, which left 69 people dead and many others seriously injured or traumatised [22]. The participants at the youth camp came from all over the country, and a significant proportion of the population therefore knew someone or had somebody in their community that was directly affected by the attacks. The terrorist attacks received massive media attention, and large memorial gatherings were organised across the entire country in the days following the attacks.

### Study design and data sources

In this study, we used data covering the entire Norwegian population gathered from the Norway Control and Payment of Health Reimbursement (KUHR) database [23]. This database contains information on healthcare consultations provided by primary care practitioners, predominantly organised under municipalities in Norway. This includes visits to GPs and emergency primary care. The purpose of the database is for practitioners to send invoices for their services directly to the state, rather than requiring patients to pay up front for later reimbursements. Although not produced for research purposes, the database has been widely used for research. In the current study, we included contacts with emergency primary care physicians. Following every contact, these professionals are required to register the contact in order to receive reimbursement from the state. In this registration, a reason for encounter must be included and categorised according to the ICPC-2 classification for primary healthcare [24]. The focus of the current study was on contacts coded as being related to psychological and/or psychiatric disorders and concerns. Data were aggregated to the day-level and anonymised prior to being retrieved from the registries.

Emergency primary care is primarily an out-of-hours service that takes in patients outside of regular business hours, or patients that are temporarily away from their place of residence and thereby unable to consult their GP. After the terrorist attacks on 22 July, the emergency primary care in Oslo had a particular responsibility to take in those patients affected by the attacks that did not have serious physical injuries [25]. They also had a central role in the provision of psychosocial follow-up in Oslo.

### Measures

#### Contacts

The main outcome of our analysis concerned daily incidents of contacts with emergency primary care, categorised as pertaining to psychological disorders or distress. The types of contacts would include consultations in person but also, e.g., telephone. These were collected retrospectively for the period 1 January 2008—31 December 2013. This covers all ICPC-2 reasons for encounter with code P [24]. In addition, the total number of daily incidents of contacts (including all reasons for encounter) were collected for the same time period. The number of daily incidents were summarised and aggregated to the weekly level to facilitate analysis.

#### Geographical proximity to the attacks

Geographical proximity to the attacks was measured by comparing contacts that had taken place in Oslo (the capital, where the bombing took place) with those in the rest of the country. Due to anonymization concerns in the registry data, it was not possible to include Hole municipality (where the shooting took place) in the measure for geographical proximity, since this municipality is small population-wise.

#### Individual characteristics

To explore whether groups of the population were affected heterogeneously, individual characteristics, i.e., the age and sex (as recorded in the registry) of patients were included in the analysis. Age was divided into 5-year age groups for anonymisation purposes. The youngest and the eldest were gathered in larger age groups (0–10 for the youngest and 86 upwards for the eldest) to ensure anonymity. Information about age and sex were generated in the registry from the patient’s social security number.

### Statistical analysis

It was an objective of the study to emulate, as far as possible, methods utilised in previous studies within the same domain in order to minimise the risk of methodological differences that could account for diverging results, when compared to other studies. Three other studies were found in which the consequences of terrorism for public health were evaluated through a time series intervention approach with Autoregressive integrated moving average (ARIMA) modelling [6, 10, 11]. This approach was therefore also selected for the current study.

The time series intervention analysis was used to estimate whether there was indeed a change in healthcare utilisation associated with the terrorist attacks. Data were aggregated to the weekly level to facilitate prediction and avoid variation in the material due to the day of the week. The weekly aggregation was done so that each week started on a Friday. This diverges from the approach chosen by other studies on the immediate aftermath of terrorism [10, 11], in which weeks were aggregated to start the day after the terrorist attacks under study. The reason for this is the difference in outcome between the studies. In the current study, the focus was on primary care contacts, which we could expect to commence already on the day of the attacks, as primary care was responsible for providing psychological care in the acute aftermath of the attacks. It was thus reasonable to start measuring the first “post-attack” week on the day of the attacks, Friday 22 July 2011. The aggregation resulted in 314 consecutive weekly observations, consisting of a variable summarising of all contacts in the given week. Since the first and last week of the time series did not add up to 7 days, these were excluded from the analysis, leaving us with 312 weekly observations.

The ARIMA model, as detailed by Cryer and Chan [26], and Hyndman and Athanasopoulos [27], was used to model the development of primary care contacts over time. The ARIMA approach enables us to take trend, seasonality and autocorrelation in the time series into account when modelling [28]. The time series was visually inspected to look for trends and seasonality before autocorrelation was calculated. The time series for all contacts with emergency primary care showed no tendency of seasonality upon visual inspection. When the ACF was calculated and plotted, however, the time series showed the tendencies of a trend.

To study the effect of the terrorist attacks on the hypothetical, unperturbed time series of healthcare contacts, we utilised intervention analysis. Intervention analysis or interrupted time series analysis as it is also called, was first introduced by Box and Tiao [29]. This is an approach that enables the introduction of external factors into time series analysis [11, 26, 28]. More specifically, it enables the modelling of the effect of an external factor on a time series’ trend and temporal development. An important challenge with this type of modelling is that it requires the estimation of when we expect to see the effect of the intervention. Based on findings from previous research on the aftermath of terror in Norway [9], we expected that the effect of the terrorist attacks would be immediate. This then led us to model the “intervention effect” as a pulse variable, which entails an immediate impact. The pulse variable is a binary variable, which is 1 in the week starting on the day of the attacks, and otherwise 0 [11, 28].

All analyses were performed in the statistical software R, using the forecast package [30] for the modelling. The auto.arima function was used for modelling, as recommended by, e.g., Schaffer et al. [28].

### Ethics

The study was evaluated by the Norwegian Regional Committee for Medical and Health Research Ethics but was not found to need ethical approval under their jurisdiction.

## Results

From 2008–2013, 555,676 contacts were registered with emergency primary care, where the reason for encounter was psychological disorders or concerns.

### The association between the terrorist attacks and consultations with emergency primary care regarding psychological concerns and disorders

**Fig. 1:**
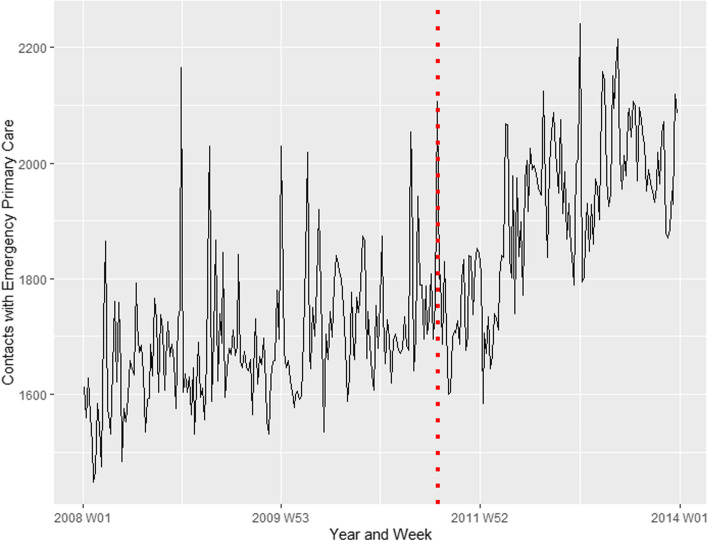
Contacts with emergency primary care for psychological disorders or concerns. Covers the entire Norwegian population from 1 January 2008 – 31 December 2013. The red dotted line represents the time of the terrorist attacks, 22 July 2011

Figure 1 shows the time series for contacts with emergency primary care for psychological disorders or concerns for the period 2008–2013. The red dotted line indicates the 22 July attacks. Based on initial visual inspections of the plot, it is difficult to decide whether there were any clear changes associated with the terrorist attacks. To formally detect whether the terrorist attacks were associated with a change in healthcare utilisation, a time series intervention analysis was conducted, the results of which are presented in Table 1. The point estimate corresponds to the increase in number of contacts in the week following the attack, compared to the modelled expected number of contacts based on characteristics of the entire time series. As we can see, the point estimates suggest that there was a statistically significant increase in contacts with emergency primary care associated with the terrorist attacks.

**Table 1:** Result of time series intervention analysis with ARIMA modelling

Dependent variable	ARIMA (p,d,q)	Pulse		
		Coef	95% CI	***p*-value**
Contacts with emergency primary care	0, 1, 2	338.92	160.58, 517.26	< .001
In Oslo only	0,1,2	21.57	-5.89, 49.03	.124
Rest of the country (Oslo excluded)	0,1,2	317.00	146.30, 487.70	< .001
Female patients only	1, 1, 2	180.12	85.23, 275.00	< .001
Male patients only	1,1,1	165.25	56.21,274.30	.003
Patients aged 0–10 only	0, 0, 0	-4.67	-12.26, 2.91	.227
Patients aged 11–15 only	1, 1, 4	4.09	-7.81, 15.98	.501
Patients aged 16–20 only	3, 1, 1	52.26	15.93, 88.59	.005
Patients aged 21–25 only	1, 1, 1	39.10	7.19, 71.00	.016
Patients aged 26–30 only	0, 1, 2	25.96	-4.26, 56.19	.092
Patients aged 31–35 only	0, 1, 1	24.60	-2.91, 52.11	.080
Patients aged 36–40 only	1, 1, 1	27.36	-0.69, 55.41	.059
Patients aged 41–45 only	1, 1, 1	51.97	23.62, 80.32	< .001
Patients aged 46–50 only	0, 1, 2	55.12	25.47, 84.78	< .001
Patients aged 51–55 only	2, 1, 1	7.91	-22.04, 37.86	.605
Patients aged 56–60 only	2, 1, 3	21.24	-3.35, 45.82	.090
Patients aged 61–65 only	0, 1, 2	12.67	-9.28, 34.62	.258
Patients aged 66–70 only	0, 1, 3	-2.31	-19.16, 14.54	.788
Patients aged 71–75 only	1, 1, 1	15.73	0.52, 30.95	.043
Patients aged 76–80 only	1, 0, 0	15.41	-2.45, 33.27	.091
Patients aged 81–85 only	3, 1, 1	5.89	-11.51, 23.29	.507
Patients aged 86 and above only	4, 0, 1	5.56	-13.44, 24.55	.566

Models based on the entire dataset, which included a time series of 312 weekly observations consisting of a variable summarizing all contacts with emergency primary care in Norway, where the reason for encounter was psychological disorder or concerns. The pulse variable was estimated using interrupted time series analysis and represents the estimated effect of the terrorist attacks; that is the estimated change in number of contacts in the week following the attacks, compared to the expected number of contacts based on the ARIMA model. The parameters p, d, q correspond to the three different parts of an ARIMA model, where p represents the number of autoregressive terms, d is the number of non-seasonal differences needed for stationarity, and q is the moving average parameter

### Contacts with emergency primary care for psychological disorders or concerns in Oslo versus the rest of the country

**Fig. 2:**
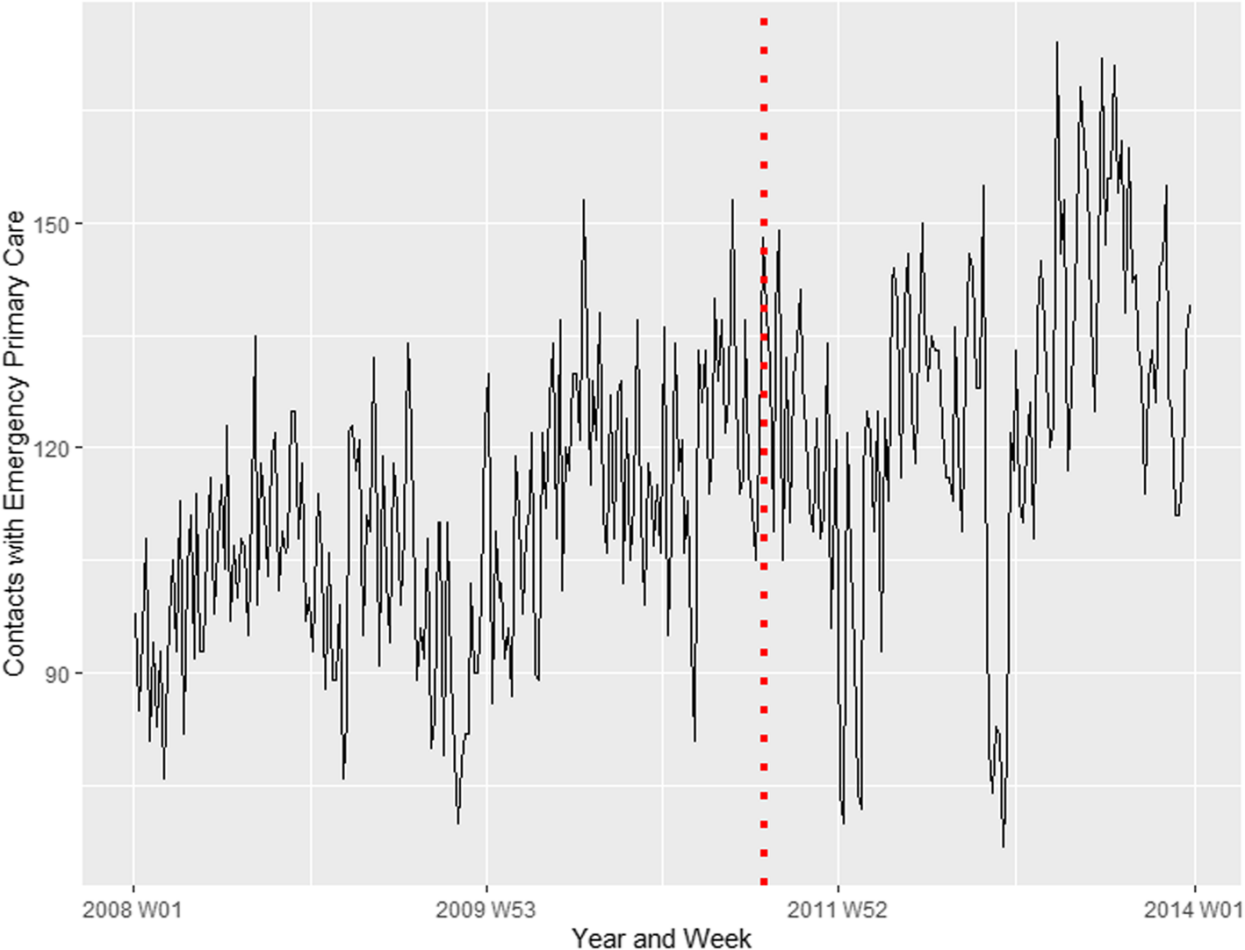
Contacts with emergency primary care in the population of Oslo for psychological disorders or concerns. Covers the time period from 1 January 2008 – 31 December 2013. The red dotted line represents the time of the terrorist attacks, 22 July 2011

**Fig. 3:**
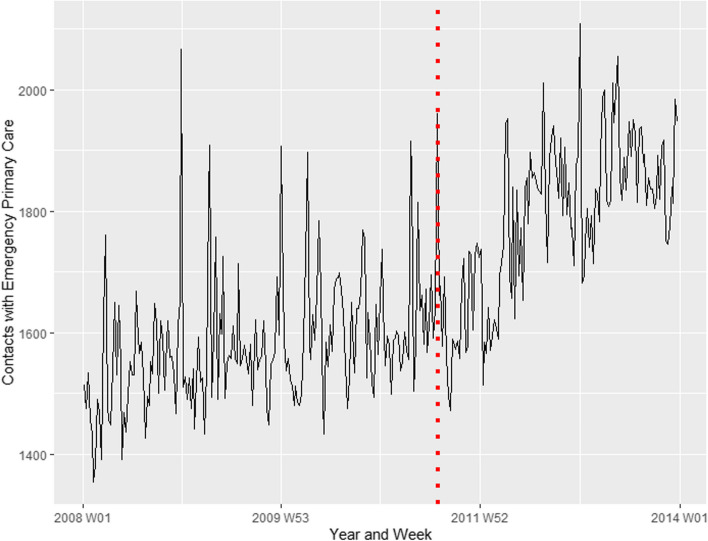
Contacts with emergency primary care in the population in the rest of Norway (Oslo excluded). Represents contacts for psychological disorders or concerns from 1 January 2008 – 31 December 2013. The red dotted line represents the time of the terrorist attacks, 22 July 2011

Of contacts with emergency primary care for psychological disorders or concerns in the study period, 36,153 took place in Oslo, while 519,523 were registered outside the capital. Figure 2 shows the time series for contacts with emergency primary care in Oslo, while Fig. 3 shows the equivalent for the rest of country. The vertical red line indicates the week of the attacks. The results for the whole country, Oslo and outside Oslo, are presented in Table 1. As is evident from the point estimates and the lack of overlap between the confidence intervals, a significant increase in contacts was estimated in the rest of the country, but not in Oslo.

### Patient groups divided according to the individual characteristics of the patient (age and sex)

**Fig. 4:**
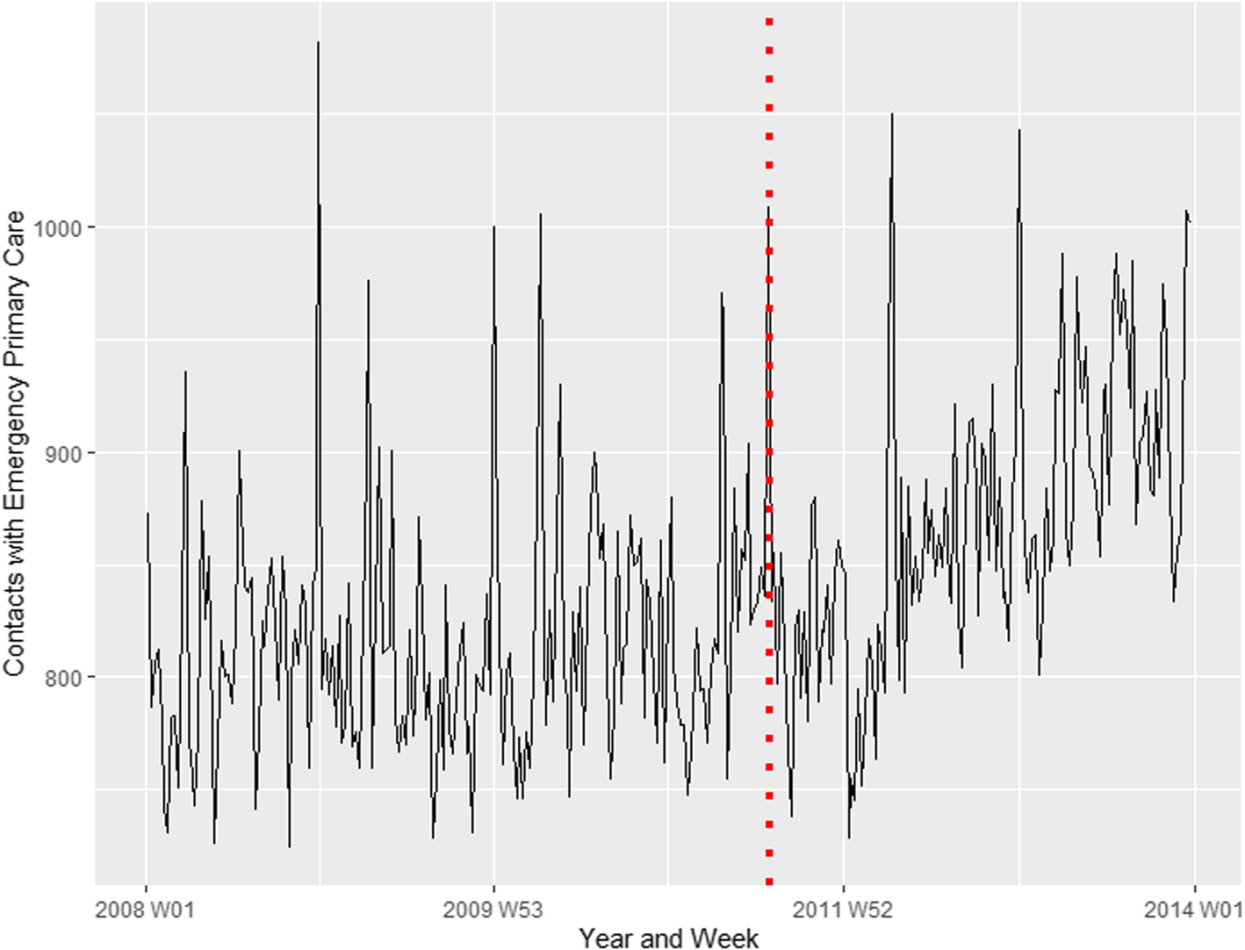
Contacts with emergency primary care in the female population. Represents contacts for psychological disorders or concerns from 1 January 2008 – 31 December 2013. The red dotted line represents the time of the terrorist attacks, 22 July 2011

**Fig. 5:**
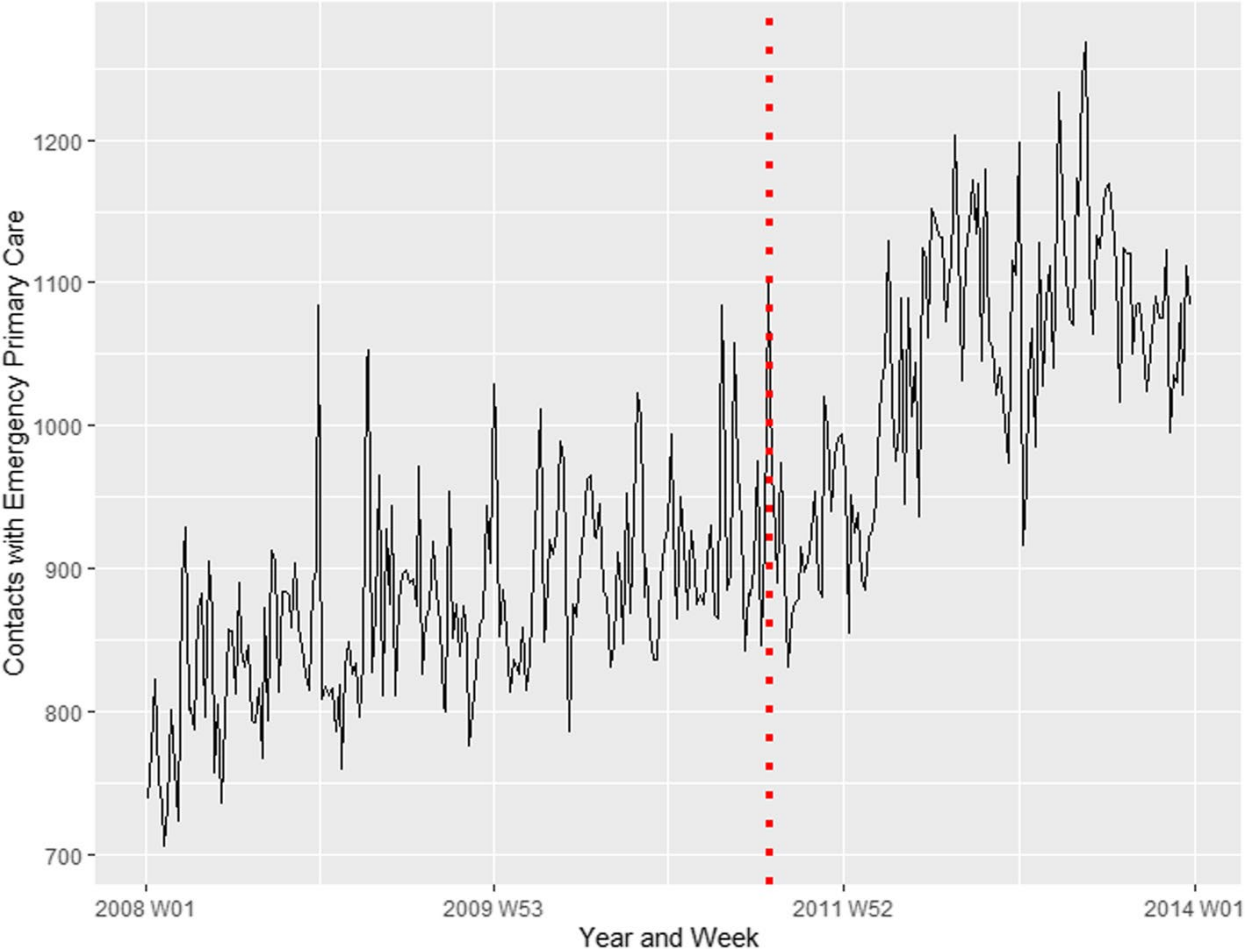
Contacts with emergency primary care in the male population. Represents contacts for psychological disorders or concerns from 1 January 2008 – 31 December 2013. The red dotted line represents the time of the terrorist attacks, 22 July 2011

Figure 4 shows the time series for contacts with emergency primary care in the female population, while Fig. 5 shows the equivalent for the male population. The vertical red lines indicate the week of the attacks. There was a significant increase in the number of contacts observed among both females and males, when the population was divided accordingly before the analysis (Table 1). Although the point estimate is slightly higher in the female population as compared to the male population, we cannot establish whether there was indeed any difference between them, given the overlapping confidence intervals.

When groups of the population divided by age were studied separately, a significant increase in contacts was found for the population aged 16–25, and 41–50 (Table 1). An increase was also observed for the age group 71–75, but here the point estimate was smaller than for the other groups, and the confidence interval runs very close to 0, which indicates more uncertainty about this latter finding.

Finally, to investigate whether the attacks were followed by changes in visits due to mental health concerns specifically, or due to changes in contacts with primary care more generally, a time series including all contacts with emergency primary care, regardless of reason for encounter, was analysed descriptively. These descriptive analyses are summarized in Additional file 1. As can be seen in Table A1 in Additional file 1, the descriptive analyses did not show any sign of an overall increase in contacts associated with the attacks. If anything, there appeared to be a decrease in the total number of contacts with emergency primary care in the week of the terrorist attacks, as compared to the weeks before and after.

## Discussion

This study contributed new insight into changes in the general population’s healthcare seeking for psychological concerns in the early aftermath of large-scale terrorist attacks using pre- and post-attack primary care data from the entire population. It used administrative data on healthcare utilisation in emergency primary care for mental health concerns, both before and after terrorist attacks. The analysis unveiled an overall increase in contacts with emergency primary care for psychological disorders and concerns in the week after the terrorist attacks on 22 July 2011 in Norway. When the population was divided into sub-groups, according to geographical closeness to the attacks, sex and age, the results did not indicate an increase in Oslo, where an attack occurred, but in the rest of the country. Furthermore, a significant increase in the number of contacts were detected for youths, young adults, and middle-aged people, but not for other age groups. Finally, an increase was found for both males and females.

Previous studies that have found reactions among indirectly affected populations when analysing data before and after terrorist attacks have used data from specialised health services and outcomes that are likely to mainly pertain to populations that had either already received mental health treatment [10, 11] or were at elevated risk for conditions of relatively low incidence [9] prior to the attacks. In a gatekeeping system, many of the patients included in studies based on data from psychiatric services will already be in specialised mental healthcare when the attack in question occurs, because healthcare data are studied short-term and referral to these services can take time. Studying data from emergency primary care, on the other hand, will to a larger extent capture contacts that are initiated acutely after the attack in question, because patients generally do not book appointments in advance, but seek help from these services acutely. Moreover, it will cover both contacts that were treated in emergency primary care without further referral as well as those that lead to a referral to specialised mental healthcare.

While this study found an overall increase in contacts for the population as a whole, the results concurrently give reason to stress the importance of distinguishing between different groups of the population, in terms of vulnerability for experiencing reactions. An important task for research is hence to identify these groups. There continues to be uncertainty regarding the mechanism through which the population’s health is affected by the occurrence of terrorist attacks. However, perceptions of proximity, including psychological proximity to the directly affected, or being in geographical proximity to attacks, have been suggested as possible explanations [2, 11, 19]. Contrary to our expectations, the geographical proximity theory could not be confirmed in the current study. Here it is important to note, though, that we examined healthcare contacts due to psychological concerns, and not psychological reactions per se. Psychological reactions do not necessarily lead to healthcare contacts. In the case of Oslo, there is a possibility that the population exerted absenteeism, assuming that the healthcare services would be overwhelmed in the immediate aftermath of the attack. Similar findings were made regarding emergency primary mental healthcare utilization during the initial phase of the Covid-19 pandemic [31]. In addition, the fact that many people across the country knew someone potentially affected by the attacks could be part of the explanation of the increases observed outside Oslo. The attack on Utøya island affected a diverse population in terms of geographical belonging, as camp participants came from all over Norway. Previous research has documented that a relatively high proportion of the population was in psychological proximity to the attacks, e.g., through reporting to have worried about the safety of loved ones, or through knowing someone directly affected by the attacks [2]. Finally, we were only able to evaluate the importance of geographical proximity in the case of Oslo, a large city, but not in the case of Hole municipality, a small community, where the Utøya attack occurred. The importance of geographical proximity might have been more elevated for the population of the smaller municipality than what would be the case in a city, but the design of the current study did not enable us to test this.

At the same time, other studies have observed reactions to terrorism very far away geographically from the attack epicenter, including in other countries [8, 10, 11], suggesting that other factors besides psychological and geographical proximity may be of importance. One such factor could be a perceived *social proximity—*that is, identifying with direct victims, although not being in any actual psychological or geographical proximity to them. This perceived *social proximity* could potentially be linked to feeling similar to those directly affected, e.g., in terms of demographic, cultural or socioeconomic characteristics, without actually knowing any victims personally. This could be an additional possible explanation for why an increase in contacts was observed among youths and young adults, as many of those directly affected in the 22 July 2011 terrorist attacks were of that age. Furthermore, it could be a possible explanation for the increase observed among middle aged individuals as these were the same age as many of the parents of the directly affected. This, however, needs to be explored in further studies. Again, there is a possibility that the results in part reflect patterns of healthcare utilisation. The fact that the increase in healthcare contacts due to psychological concerns pertained to certain age groups only does not necessarily mean that the same applied for psychological reactions. Some age groups might indeed have been less likely to access emergency primary care themselves, e.g., children and the elderly.

When separate analyses were conducted for males and females, an increase was found for both groups. Often, the female sex has been reported as a risk factor for developing stress reactions, such as post-traumatic stress disorder [32]. However, not many studies have studied sex differences in health outcomes in the general population after terrorism. Our findings also highlight the importance of not underestimating stress reactions in the male population in the aftermath of terror. Studying contacts with primary emergency care related to mental health could also be particularly relevant when considering the needs of the male population in this regard. In Norway, men are consistently overrepresented in contacting emergency primary care services for psychological disorders or concerns, whereas women are highly overrepresented in seeking help with general practitioners for the same concerns [33]. Studying emergency primary care services specifically could therefore be important in order to capture reactions in both sexes. Nonetheless, given that women to a larger extent tend to seek help with their GP rather than emergency primary care for psychological concerns, our study may have failed to identify potential sex differences in overall healthcare seeking for psychological concerns.

Finally, characteristics of the attacks could also be an explanatory factor for why reactions are observed in certain contexts, but not in others. Byrne et al. [1] discuss whether the lack of mental health reactions observed in the general population in their study could be ascribed to characteristics of the attack studied, which was the March 2019 mosque shootings in Christchurch, New Zealand. More specifically, the attack in their case targeted a minority population in the country. Byrne et al. [1] link this to the extent to which populations empathise or sympathise with the direct victims. An alternative explanation could be linked to perceptions of threat, including whether members of the population perceive that they themselves or someone close to them are unsafe due to the attack [4]. The attacks studied in the current paper targeted members of the majority population, which could imply that larger segments of the population felt threatened.

### Strengths and limitations

A major strength of the current study is that it included data on emergency primary care utilisation from the entire population before and after a terrorist attack. This enabled the investigation of potential changes in the wake of the attacks without the selection and recall biases that may hamper survey-based research, or the lack of comparison data that characterises cross-sectional studies. Furthermore, previous studies using registry or administrative data on healthcare utilisation or diagnoses have often only included data from the entire population without information on potential differences according to geographic closeness to the attacks, age and sex. Still, why certain groups appear to be affected more strongly is discussed widely in the literature. The ability to differentiate these groups is a strength of the current study. At the same time, a limitation of this study is that the data utilised were not collected for research purposes. This means that we were not able to isolate factors such as direct exposure to the attacks. Since we used aggregated data, we could also not investigate individual factors such as, e.g., previous history of mental illness. This is a relevant factor given that individuals with previous mental illness at the time of the attack appear to be more vulnerable to experiencing psychological reactions after the event [34]. Furthermore, we could not directly evaluate the importance of psychological proximity, including knowing someone directly affected, which has been found to be associated with increased distress in previous studies [2]. Finally, how contacts are coded by healthcare personnel could potentially lead to error in the data; however, we have no reason to assume that this follows a systematic pattern.

It should also be noted that emergency primary care services will provide consultations for certain psychological or psychiatric disorders more than others. The type of mental health concerns treated at emergency primary care facilities are typically of an acute character, given that these are out-of-hours services that take in patients whose disorder or concern is so serious that treatment cannot wait for them to get in touch with their GP. Strand et al. [9], e.g., found an immediate increase in episodes of schizophrenia/psychosis after the terrorist attacks. These are conditions that will typically require acute treatment, and it could therefore be that our findings in part reflect those of Strand et al. [9]. If this is the case, the current study still provides important information about where in the system health concerns end up needing treatment – in this case, in the primary care services. Furthermore, the current study gives information about reactions in diverse groups of the population, divided according to age, sex and geographical closeness to the attacks. To our knowledge, this has not been assessed earlier.

It should be noted that these data only capture mental health reactions that lead to help-seeking behaviour. There could be reactions in the population that are not captured through this study, because persons did not seek help from the healthcare system, or because the healthcare system does not have sufficient resources to take in everyone. The latter can be particularly relevant in a crisis situation, such as the immediate aftermath of a terrorist attack. Particularly in Oslo, the emergency primary care services had a central role in taking care of individuals affected by the bombing yet with less severe physical injuries or none at all. It is reasonable to assume that this implied a specific strain on the clinics in Oslo, which could lead to a lack of resources to take in other patients. At the same time, the smaller and uncertain estimated change in Oslo could suggest that the observed effects were not due to directly affected individuals only. Furthermore, those directly affected at the government quarters in Oslo received immediate psychosocial assistance through an extraordinary service, organised outside regular services at emergency primary care [35].

An important dimension when considering reactions after terrorist attacks is that of time. Hypothesising a priori what we expect the impact of the intervention to be on the times series is an important, although sometimes overlooked, aspect of ARIMA modelling [28]. Based on findings from previous literature [9], we hypothesised that any change in the number of contacts would be immediate. The study can therefore not say anything about development with more time or any potential long-term effects. However, as time passes, it becomes increasingly difficult to isolate whether the effects measured could be ascribed to the attacks. For this reason, the modelling of effects at longer time spans, with the notable exception of child and maternal health, which can arguably be isolated in a longer time span [6], is challenging.

The study utilised an analytical method that has previously been used in similar studies, and this was done to increase the possibilities for comparison of results, facilitating the isolation of whether any diverging findings are due to different study contexts or different methods utilised. Furthermore, the study utilised aggregated data, so that the method can be replicated in other settings where data availability from registries and administrative databases may be limited to aggregated data only. This is considered an important strength of the current study. A novelty of the approach utilised was to study disaggregated data, according to geographical proximity to the attacks, age, and sex. While this provided valuable information about how the terrorist attacks affected groups of the population differently, this disaggregation came with certain limitations that should be noted. First, the 22 July 2011 terrorist attack occurred in two separate locations: the capital of Oslo and Utøya island in Hole municipality, which both would have been relevant to consider when evaluating the importance of geographical proximity. However, the analysis could only be conducted in the case of Oslo. The utilization of registry data in the current study entailed that the data were anonymous, not only in the presentation of results, but also for the team of researchers conducting the analysis. The population size of Hole municipality was 6,140 in 2011 [36]. This meant that Hole was too small, both in terms of population size and the number of daily or weekly consultations to allow for separate analysis of that municipality. In addition, Hole municipality does not have its own emergency primary care unit but is cooperating with other municipalities in providing this service, which also would have made it challenging to single out consultations from Hole only. For these reasons Hole could not be considered when the importance of geographical proximity was evaluated. Due to the small size of the population, however, any potential increase in the number of consultations in Hole due to geographical proximity to the Utøya attack, would be too small to affect the results covering the entire population, when being compared to Oslo. Second, the disaggregated analysis of different age groups should be interpreted with care, given the disaggregation of the material into a fairly high number of sub-groups and the partly overlapping confidence intervals.

The findings from the current study are not necessarily transferable to all other contexts of terrorism. As warned by Chatignoux et al. [13], for instance, other trajectories may be observed, e.g., in cases of repeated attacks, where stress levels in the population may be elevated over time or develop according to a habituation response [21]. Furthermore, the findings of this study must be understood in the context of primary care in a gatekeeping healthcare system. Still, the findings of this article are important contributions to existing literature in questioning whether pathology should be expected in the population at large after terrorist attacks and establishing the need to differentiate between different subgroups.

## Conclusion

The 22 July 2011 attacks in Norway were followed by an immediate increase in the number of contacts with emergency primary healthcare due to mental health concerns. At the same time, whereas an increase was observed for the population as a whole, the results also indicated that certain groups were affected more than others. These findings highlight the need to consider the population as a heterogeneous entity in the aftermath of terrorism. Whereas previous research has questioned whether characteristics of the attacks in question are important for deciding whether the general, indirectly affected population will experience pathology post-terror, the current study stresses the importance of also taking demographic factors into account. Reactions in the general population in the aftermath of collective violence will not necessarily lead to illness. Among certain groups however, reactions can lead to pathology. The current study shows that primary care services need to be prepared to handle these healthcare needs. This knowledge is important for future planning of healthcare responses after terrorism. Future research should focus on further identifying groups of the population vulnerable for developing reactions post-terror, and also explore the role of primary care further, e.g., through studying healthcare seeking with GPs.

### Supplementary Information

**Additional file 1.** All contacts with Primary Emergency Care: Any Reason for Encounter.

## Data Availability

The datasets used and/or analyzed are available from the KUHR database (Available from: https://www.helsedirektoratet.no/tema/statistikk-registre-og-rapporter/helsedata-og-helseregistre/kuhr).
