# Supplementary material for: The short-term impact of terrorism on public mental health: an emergency primary care approach
Source: BMC Public Health. 2023 Nov 24;23:2325. doi: 10.1186/s12889-023-17240-z (PMC10668408; doi:10.1186/s12889-023-17240-z)
Supplement: Supplementary file 1 — Additional file 1. All contacts with Primary Emergency Care: Any Reason for Encounter. [file 12889_2023_17240_MOESM1_ESM.docx]

# Additional file 1: All contacts with Primary Emergency Care: Any Reason for Encounter

# Contacts with Emergency Care – All Reasons for Encounter


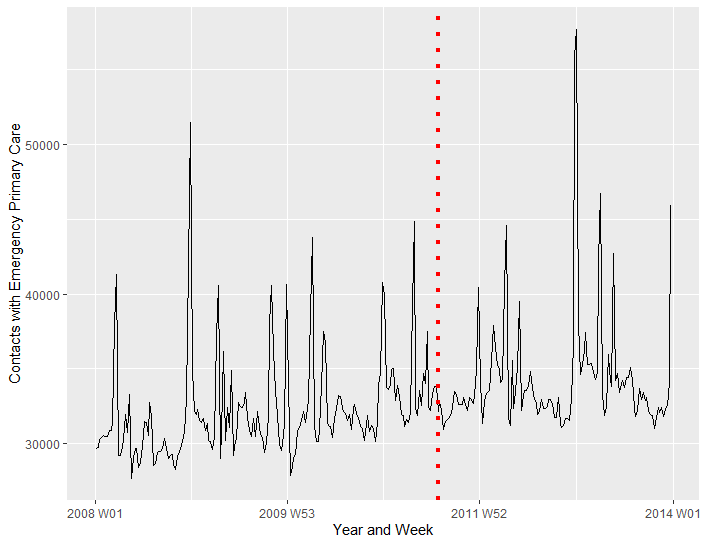


The graph shows the time series for all contacts with Emergency Primary Care, regardless of reason for encounter. Again, the red, dotted vertical line illustrates the week of the terrorist attack. When inspecting the graph visually, no sign of any increase in association with the attacks can be detected. The table below show the numbers of weekly contacts with emergency primary care in the weeks before and after the attacks. For comparison, the same period the year before and after are also included. There is no indication of any increase in the number of contacts the week of the attack.

Table A1 All contacts with emergency primary care in the summers of 2011 (the year of the attack), 2010 (the year before the attack) and 2012 (the year after the attack)

|  | All contacts | Oslo only | | Outside Oslo only | Male patients only | Female patients only |
| --- | --- | --- | --- | --- | --- | --- |
| 01. - 07.07.2011 | 33254 | 2827 | | 30427 | 15811 | 17443 |
| 08. - 14.07.2011 | 33831 | 2938 | | 30893 | 15658 | 18173 |
| 15. - 21.07.2011 | 33805 | 2827 | | 30978 | 15640 | 18165 |
| 22. - 28.07.2011* | **32125** | **2741** | | **29384** | **14994** | **17131** |
| 29.07. - 04.08.2011 | 32825 | 2777 | | 30048 | 15418 | 17407 |
| 05. - 11.08.2011 | 32239 | 2651 | | 29588 | 15256 | 16983 |
| 12. - 18.08.2011 | 30921 | 2565 | | 28356 | 14630 | 16291 |
| 19. - 25.08.2011 | 31452 | 2716 | | 28736 | 14956 | 16496 |
| 26.08. - 01.09.2011 | 31625 | 2735 | | 28890 | 14854 | 16771 |
|  |  |  | |  |  |  |
| Same time period year prior to attacks | | |  |  |  |  |
| 02. - 18.07.2010 | 32321 | 2793 | | 29528 | 15161 | 17160 |
| 09. - 15.07.2010 | 33224 | 3111 | | 30113 | 15707 | 17517 |
| 16. - 22.07.2010 | 33157 | 2944 | | 30213 | 15432 | 17725 |
| 23. - 29.07.2010 | 32435 | 2928 | | 29507 | 15094 | 17341 |
| 30.07. - 05.08.2010 | 32124 | 2914 | | 29210 | 15000 | 17124 |
| 06. - 12.08.2010 | 32038 | 2902 | | 29136 | 14824 | 17214 |
| 13. - 19.08.2010 | 31540 | 2781 | | 28759 | 14710 | 16830 |
| 20. - 26.08.2010 | 31906 | 2697 | | 29209 | 14970 | 16936 |
| 27.08. - 02.09.2010 | 30925 | 2730 | | 28195 | 14553 | 16372 |
|  |  |  | |  |  |  |
| Same time period year after attacks | |  | |  |  |  |
| 06. - 12.07.2012 | 34826 | 3152 | | 31674 | 16427 | 18399 |
| 13. - 19.07.2012 | 33671 | 3024 | | 30647 | 15818 | 17853 |
| 20. - 26.07.2012 | 32939 | 2821 | | 30118 | 15278 | 17661 |
| 27.07 - 02.08.2012 | 32769 | 2798 | | 29971 | 15212 | 17557 |
| 03. - 09.08.2012 | 31966 | 2668 | | 29298 | 15014 | 16952 |
| 10. - 16.08.2012 | 32115 | 2540 | | 29575 | 15375 | 16740 |
| 17. - 23.08.2012 | 32949 | 2683 | | 30266 | 15575 | 17374 |
| 24. - 30.08.2012 | 32328 | 2685 | | 29643 | 15431 | 16897 |
| 31.08. - 06.09.2012 | 32338 | 2979 | | 29359 | 15283 | 17055 |

*the week of the terrorist attack.
